# The Diuretic Torasemide Does Not Prevent Aldosterone-Mediated Mineralocorticoid Receptor Activation in Cardiomyocytes

**DOI:** 10.1371/journal.pone.0073737

**Published:** 2013-09-09

**Authors:** Basile Gravez, Antoine Tarjus, Ruben Jimenez-Canino, Soumaya El Moghrabi, Smail Messaoudi, Diego Alvarez de la Rosa, Frederic Jaisser

**Affiliations:** 1 INSERM Unité 872, Université Pierre et Marie Curie, Team 1, Centre de Recherche des Cordeliers, Paris, France; 2 Department of Physiology and Institute of Biomedical Technologies, Universidad de La Laguna, Tenerife, Spain; 3 Centre d’ Investigation Clinique, Institut Lorrain du Coeur et des Vaisseaux, Centre Hospitalier Universitaire de Brabois, Vandoeuvre-lès-Nancy, France; I2MC INSERM UMR U1048, France

## Abstract

Aldosterone binds to the mineralocorticoid receptor (MR) and exerts pleiotropic effects beyond enhancing renal sodium reabsorption. Excessive mineralocorticoid signaling is deleterious during the evolution of cardiac failure, as evidenced by the benefits provided by adding MR antagonists (MRA) to standard care in humans. In animal models of cardiovascular diseases, MRA reduce cardiac fibrosis. Interestingly diuretics such as torasemide also appear efficient to improve cardiovascular morbidity and mortality, through several mechanisms. Among them, it has been suggested that torasemide could block aldosterone binding to the MR. To evaluate whether torasemide acts as a MRA in cardiomyocytes, we compared its effects with a classic MRA such as spironolactone. We monitored ligand-induced nuclear translocation of MR-GFP and MR transactivation activity in the cardiac-like cell line H9C2 using a reporter gene assay and known endogenous aldosterone-regulated cardiac genes. Torasemide did not modify MR nuclear translocation. Aldosterone-induced MR transactivation activity was reduced by the MRA spironolactone, not by torasemide. Spironolactone blocked the induction by aldosterone of endogenous MR-responsive genes (Sgk-1, PAI-1, Orosomucoid-1, Rgs-2, Serpina-3, Tenascin-X), while torasemide was ineffective. These results show that torasemide is not an MR antagonist; its association with MRA in heart failure may however be beneficial, through actions on complementary pathways.

## Introduction

The use of loop diuretics such as furosemide and torasemide is classically included in the therapeutic arsenal for the symptomatic treatment of congestive heart failure. The TOrasemide In Congestive heart failure trial (TORIC), showed that torasemide had more beneficial effects on mortality and morbidity of patients with CHF than furosemide [1]. Part of the benefit may be related to the different pharmacokinetic properties of torasemide, such as the longer half-life, the longer duration of action and the higher bioavailability as compared to furosemide. However it has also been suggested that torasemide has pharmacodynamic properties beyond its loop diuretic effect. Indeed, torasemide has specific vascular effects as compared to furosemide: it inhibits angiotensin II (AngII)-induced vasoconstriction in the aorta of Spontaneously Hypertensive Rats [2] as well as AngII-stimulated vascular smooth muscle cell growth [3]. Torasemide also inhibits thromboxane A2-induced vasoconstriction in isolated canine artery [4]. Furthermore, a possible blockade of mineralocorticoid receptor binding of aldosterone (called anti-aldosteronergic effect in the initial paper) has been reported for torasemide by the group of Uchida in 1991: administration of a relatively high dose of torasemide significantly inhibited in vivo binding of aldosterone to its receptor in the cytoplasmic fraction of rat kidney homogenates, whereas furosemide had no effect [5]. This results led to the hypothesis that torasemide may also act as a mineralocorticoid receptor antagonist.

Indeed, inappropriate mineralocorticoid signaling has been shown to play an important role in the progression of cardiovascular (CV) disease. Aldosterone (Aldo) is a main regulator of renal sodium reabsorption with an overall effect on volemia and blood pressure. Aldo binds to the mineralocorticoid receptor (MR), a transcription factor of the nuclear receptor family present in the kidney and also in non-epithelial cells [6]. New extra-renal pathophysiological effects of this hormone have been characterized, extending its actions to the CV system and inappropriate MR activation has been shown to promote cardiac fibrosis in experimental models [7]. The Randomized Aldactone Evaluation Study (RALES) [8], the Eplerenone Post–Acute Myocardial Infarction Heart Failure Efficacy and Survival Study (EPHESUS) [9] and Eplerenone in Mild Patients Hospitalization and Survival Study in Heart Failure (EMPHASIS-HF) [10] clinical trials have demonstrated that the addition of MR antagonists to standard care markedly reduced the overall and cardiovascular mortality in patients with left ventricular systolic dysfunction and mild or severe symptoms of chronic heart failure (HF) or with signs of HF after acute myocardial infarction. The beneficial effects of MR antagonists in HF are associated with a reduction of cardiac fibrosis [7].

In patients with chronic HF, torasemide has been reported to reduce myocardial fibrosis [11]–[13]. As this effect was not observed in furosemide-treated patients, the ability of torasemide to act on myocardial fibrosis might be related to interference with profibrotic factors such as aldosterone and AngII.

This putative anti-aldosterone/anti-mineralocorticoid receptor property of torasemide has potential therapeutic outcomes in the treatment of HF. From a clinical point of view, it is therefore important to determine whether torasemide and spironolactone, the classical mineralocorticoid receptor antagonist (MRA), have similar targets and whether these drugs should be associated to potentiate their efficacy in the treatment of HF. The aim of this study was to analyze whether torasemide acts as a MRA in cardiomyocytes, in comparison with spironolactone.

## Materials and Methods

### Ligand-induced MR Nuclear Translocation Assay

COS-7 is a fibroblast-like cell line derived from monkey kidney tissue classically used for transactivation assays due to the absence of endogenous expression of MR or the related glucocorticoid receptor (GR) [14]. COS-7 cells were obtained from the American Type Culture Collection and grown in DMEM supplemented with 10% FBS. Cells were transiently transfected with a functional fluorescent variant of the mouse MR (MR-147-GFP) [15] using jetPRIME (Polyplus Transfection, Illkirch, France) according to the manufactureŕs instructions. At the time of transfection, cells grown on coverslips were transferred to medium containing charcoal-stripped serum (Lonza, Barcelona, Spain). Forty-eight hours after transfection, cells grown on coverslips were placed under an Olympus Fluoview FV1000 confocal microscope with an imaging chamber pre-heated to 37°C. For imaging experiments, culture medium was substituted by extracellular saline (10^−3^ mol/liter: NaCl, 137; KCl, 4; CaCl_2_, 1.8; MgCl_2_, 1; glucose, 10; HEPES, 10; pH 7.4). Cells were treated or not with 10^−8^ M aldosterone, 10^−6^ M torasemide (Sigma, St. Louis, MO) and 10^−6^ M spironolactone (Sigma, St. Louis, MO) and images were acquired every 2 minutes (min) for one hour. Total and nuclear fluorescence intensity was analyzed frame by frame in individual cells using the manufactureŕs software (Olympus). Data processing and curve fitting were performed using Igor Pro (Wavemetrics, Lake Oswego, OR).

### H9C2-MR Cardiomyocyte Transactivation Assay

H9C2-MR cells is a clonal cell line of cardiomyocytes derived from the embryonic rat ventricle that stably expresses the MR [16]. H9C2-MR cells (kindly provided by A Fejes-Toth) were stably transfected with a MMTV-Luc reporter construct [17] (kindly provided by H. Richard-Foy) using lipofectamine 2000 as previously described [18]. Clone 23, thereafter referred as H9C2-MR/MMTV-Luc, was selected for further transactivation studies.

H9C2-MR/MMTV-Luc cells were grown in DMEM/F12 medium supplemented with 10% fetal bovine serum plus antibiotic (penicillin-streptomycin; Invitrogen, Carlsbad, CA). 24 h before treatment, cells were grown in steroid-free medium. Cells were then treated for 24 h with aldosterone, spironolactone, the GR antagonist RU 38486 (10^−6^ M; Sigma) or torasemide, alone or in combination. Luciferase activity was assayed according to the manufactureŕs instructions (Dual-Light® Luciferase Assay; Applied Biosystems).

### Gene Expression in H9C2-MR Cells

H9C2-MR cells were treated with various concentrations (as indicated in figure legends) of aldosterone, torasemide and/or spironolactone and the expression of the MR-modulated genes was measured using quantitative RT-PCR for Serum- and glucocorticoid-inducible kinase-1 (Sgk-1), Orosomucoid-1, Serpina-3, Plasminogen Activator Inhibitor-1 (PAI-1), Tenascin-X and Regulator of G protein signaling-2 (Rgs-2).

### Quantitative RT- PCR

Total RNA was extracted from cells (4 to 8 wells per condition) using TRIZOL® reagent (InVitrogen), according to the manufacturer’s protocol, and DNase-treated. Reverse transcription was performed with 2 µg of total RNA, random primers (InVitrogen) and Superscript II reverse transcriptase (InVitrogen). Transcripts levels were analyzed by real time PCR in an iCycler iQ apparatus (Bio-Rad Laboratories, Marnes La Coquette, France) with SYBR Green I detection. The reactions were performed in duplicate for each sample using a qPCR MasterMix Plus SYBR with fluorescein (Eurogentec, Angers, France) with 5 10^−7^ M of each sense and antisense primer and 6 ng of cDNA in 15 µL total volume. The thermal cycling parameters were: initial denaturation at 95°C for 10 min, followed by 40 cycles at 95°C for 15 sec and 60°C for 1 min. Relative expression of the mRNA was quantified using the equation described by M.W. Pfaffl [19]: ratio = (E_target_)^∧Cttarget(mean control-sample)/(^E_ref_)^∧Ctref(mean control-sample)^. The mRNA levels were normalized for β-actin mRNA in samples obtained from cultured cells. Values in control conditions were set as 1 for each gene, and fold changes are provided on the figures. The sequences of the specific primers are detailed in Table S1.

### Statistical Analysis

Results are provided as mean ± SEM. Differences between experimental conditions were tested by the Kruskal-Wallis One Way Analysis of Variance on Ranks test. The Newman-Keuls posthoc test was used to adjust for multiple comparisons (SigmaPlot V.11.0 software). Values of *p*<0.05 were considered statistically significant.

## Results

### Effect of Torasemide on MR Translocation into the Nucleus

**Figure 1:**
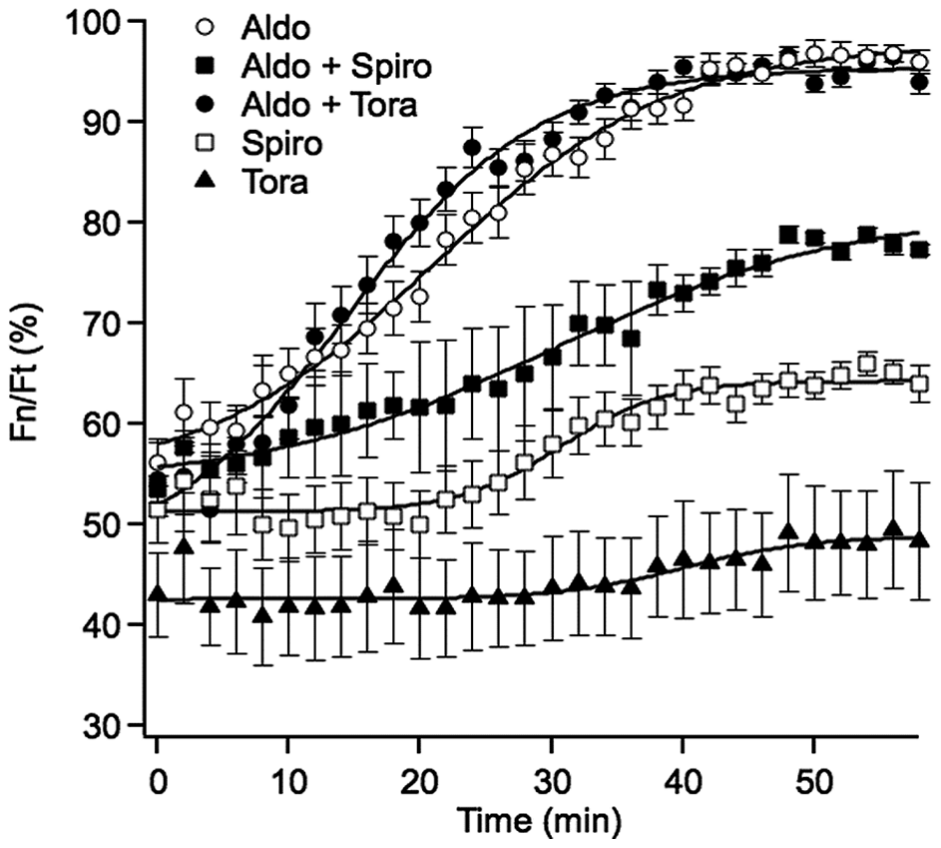
In vivo kinetics of nuclear translocation of MR in COS-7 cells. COS-7 cells transfected with GFP-MR were treated with 10^−8^ M aldosterone (Aldo), 10^−8^ M aldosterone +10^−6^ M torasemide (Aldo+ Tora), 10^−8^ M aldosterone +10^−6^ M spironolactone (Aldo+Spiro), 10^−6^ M spironolactone (Spiro) or 10^−6^ M torasemide (Tora), starting at time 0. Individual points represent the average percentage fluorescent intensity of the nucleus vs. total cellular fluorescence (Fn/Ft) measured in individual cells over the indicated period of time (± SE, n = 16). Data points were fitted to a sigmoid curve.

MR is a nuclear transcription factor. Upon ligand binding, MR shuttles from its cytoplasmic localization into the nucleus. We first analyzed the kinetics of ligand-induced MR translocation in COS-7 cells transiently transfected with the previously described MR-147-GFP functional fluorescent variant of MR [15]. It has previously been shown that under non-stimulated conditions, MR is present both in the cytoplasm and the nucleus, but addition of aldosterone induces nuclear accumulation of the receptor [20]. In cells treated with 10^−8^ M aldosterone, cytosolic MR-GFP initiated its translocation to the nucleus within minutes, with a half-maximal translocation time of approximately 18 minutes (Figure 1). In cells treated with 10^−6^ M torasemide alone (without aldosterone) no translocation of the MR-GFP was detected. Spironolactone alone (10^−6^ M), however, induced MR translocation but with a much slower rate than aldosterone alone (Figure 1), consistently with previously published reports [20].

Simultaneous addition of 10^−6^ M torasemide and 10^−8^ M aldosterone did not change the kinetics of aldosterone-induced MR-GFP nuclear translocation while spironolactone (10^−6^ M) slowed down aldosterone-induced MR nuclear translocation (Figure 1), indicating that torasemide and spironolactone behave differently, regarding aldo-induced MR nuclear translocation.

### Effect of Torasemide on Aldosterone–mediated MR Transactivation Activity

**Figure 2:**
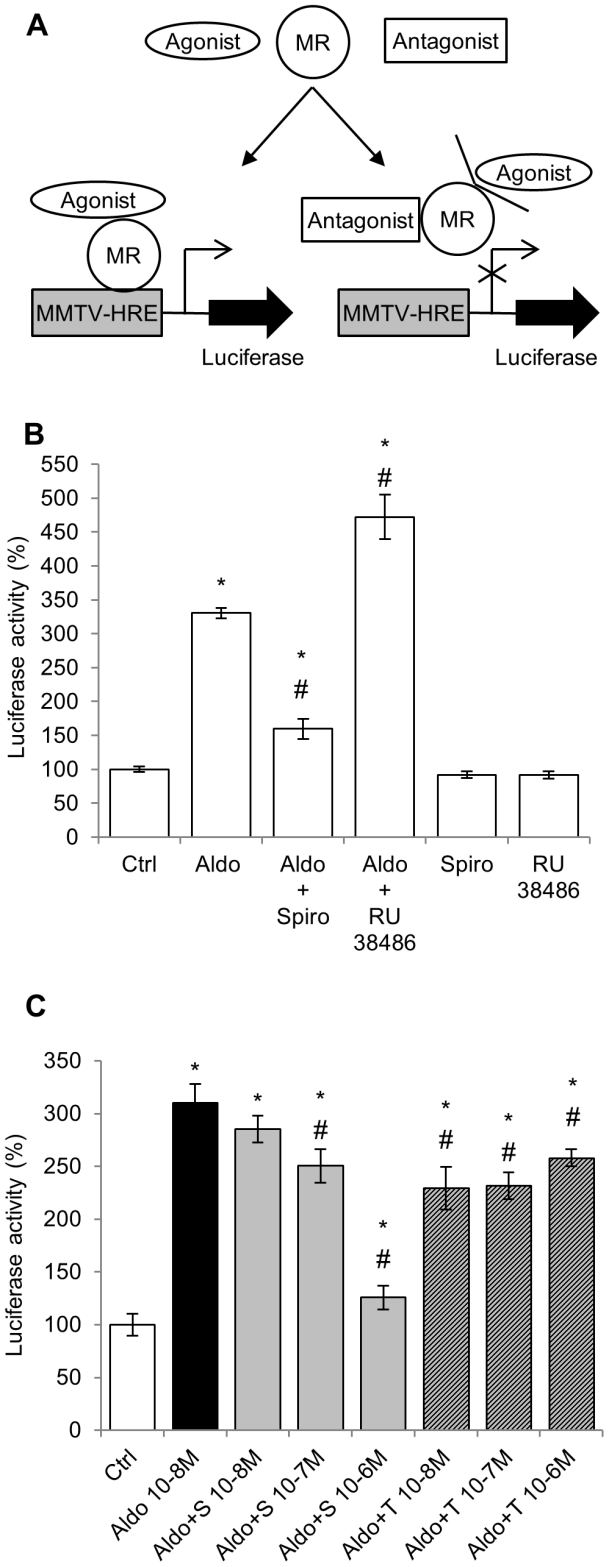
Effect of Torasemide on ligand-dependent transactivation activity of MR in H9C2-MR cells. A: The MMTV promoter contains Hormone Response Elements (HRE); the promoter sequence was fused to the Luciferase coding sequence. This construct was transfected into H9C2-MR cells. Upon binding of the aldosterone-MR complexes on HRE, luciferase is transcribed and light emission is enhanced, assessing MR transactivation activity. In the presence of an MR antagonist, binding of aldosterone to MR is prevented and luciferase expression is abolished. B: 10^−8^ M aldosterone (Aldo) increased MR transactivation activity (luciferase activity) which was prevented by a 100-fold excess of the MR antagonist spironolactone (Spiro; 10^−6^ M) but not by the GR antagonist RU 38486 (RU 38486; 10^−6^ M). Each antagonist alone has no effect. Mean ± SEM (n = 8). * *p*<0.05 *vs* control (Ctrl); # *p*<0.05 *vs* aldosterone. C: 10^−8^ M aldosterone (Aldo) increased MR transactivation activity, which was fully inhibited by the MR antagonist spironolactone (S) at 10-6 M. Torasemide (T) has a slight antagonist effect independent of its concentration. Mean ± SEM (n = 4). *p<0.05 *vs* control (Ctrl); # *p*<0.05 *vs* aldosterone.

A transactivation assay was set up in the rat H9C2-MR cardiomyocytes cell line expressing moderate levels of rat MR [16]. The system allows rapid and efficient screening of MR-dependent transactivation, using a MMTV promoter construct that includes Hormone Response Elements leading to luciferase expression upon binding of MR to the Hormone Response Elements. In the absence of MR activation, luciferase is not expressed while upon MR activation, luciferase activity is expressed and can be easily detected (Figure 2A). This widely used reporter system allows efficient analysis of molecules with agonist/antagonist activity on the MR.

Addition of 10^−8^ M aldosterone induced a 3.5 fold increase in luciferase activity which was fully inhibited by the MR antagonist spironolactone (10^−6^ M) but not by the GR antagonist RU 38486 (10^−6^ M) indicating specific MR activation by aldosterone in this cellular context (Figure 2B). Spironolactone or RU38486 alone have not effects (Figure 2B). Several concentrations of spironolactone or torasemide were then tested for their inhibitory activity of the aldosterone-mediated MR activation (Figure 2C). 10^−6^ M spironolactone fully inhibited the MR transactivation induced by 10^−8^ M aldosterone while torasemide has only a weak inhibitory effect that was concentration-independent. In order to unmask a potential inhibitory effect in the presence of a lower aldosterone concentration, the effects of spironolactone and torasemide were also tested in the presence of 10^−9^ M aldosterone. In these conditions, torasemide has no inhibitory effect at difference with a dose-dependent antagonist activity of spironolactone (Figure S1).

### Effect of Torasemide on MR-mediated Endogenous Gene Expression in H9C2-MR Cells

**Figure 3:**
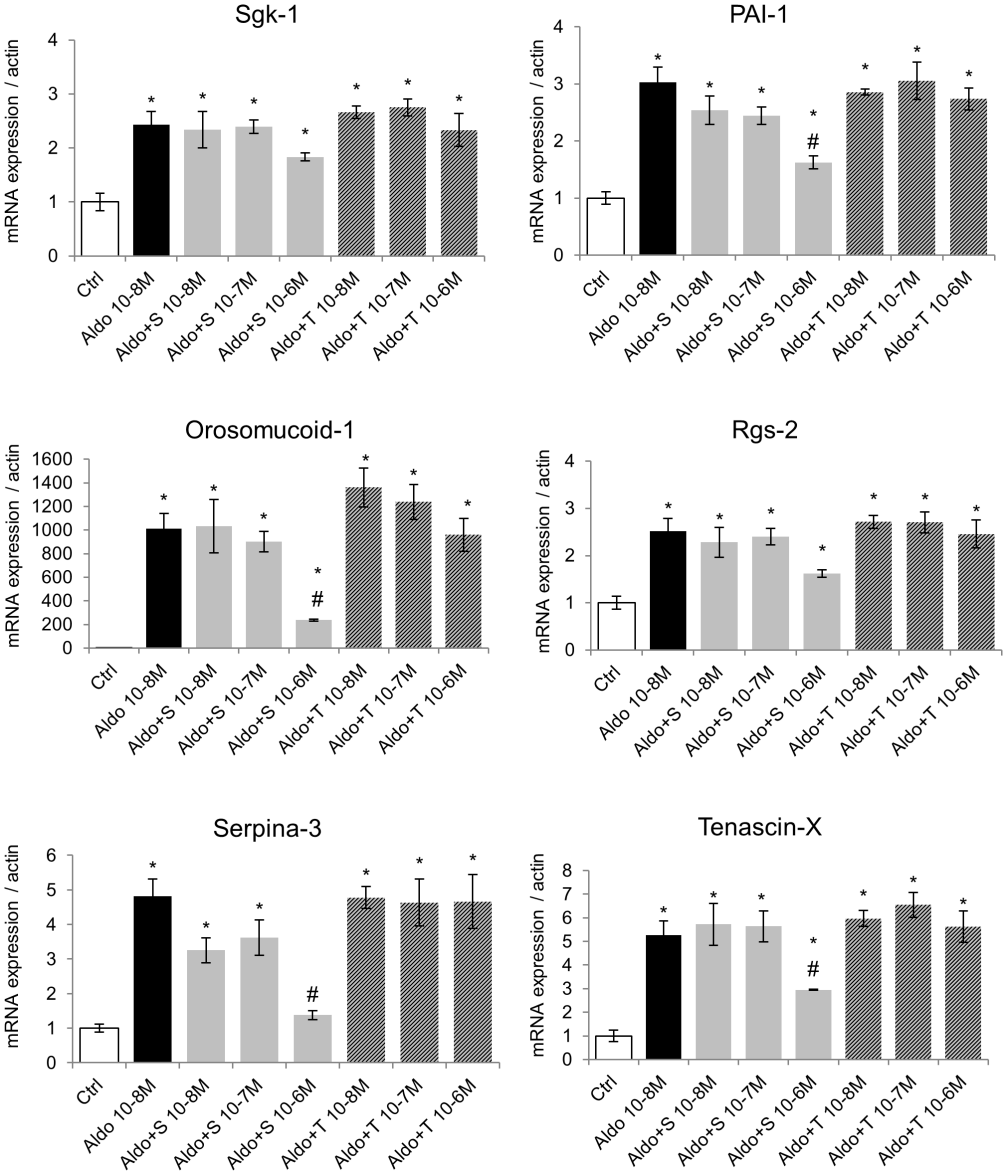
Torasemide does not act as a MR antagonist for the regulation of endogenous genes in H9C2-MR cells in the presence of 10^−8^ M aldosterone. 10^−8^ M aldosterone (Aldo) increased expression of the aldosterone-targets genes Sgk-1, PAI-1, Orosomucoid-1, Rgs-2, Serpina-3 and Tenascin-X. Addition of increasing doses of spironolactone (A+S) inhibited aldosterone-induced gene expression. In contrast, increasing concentrations of torasemide (A+T) had no antagonistic effect. Mean ± SEM (n = 4). *p<0.05 *vs* control (Ctrl); # *p*<0.05 *vs* aldosterone.

**Figure 4:**
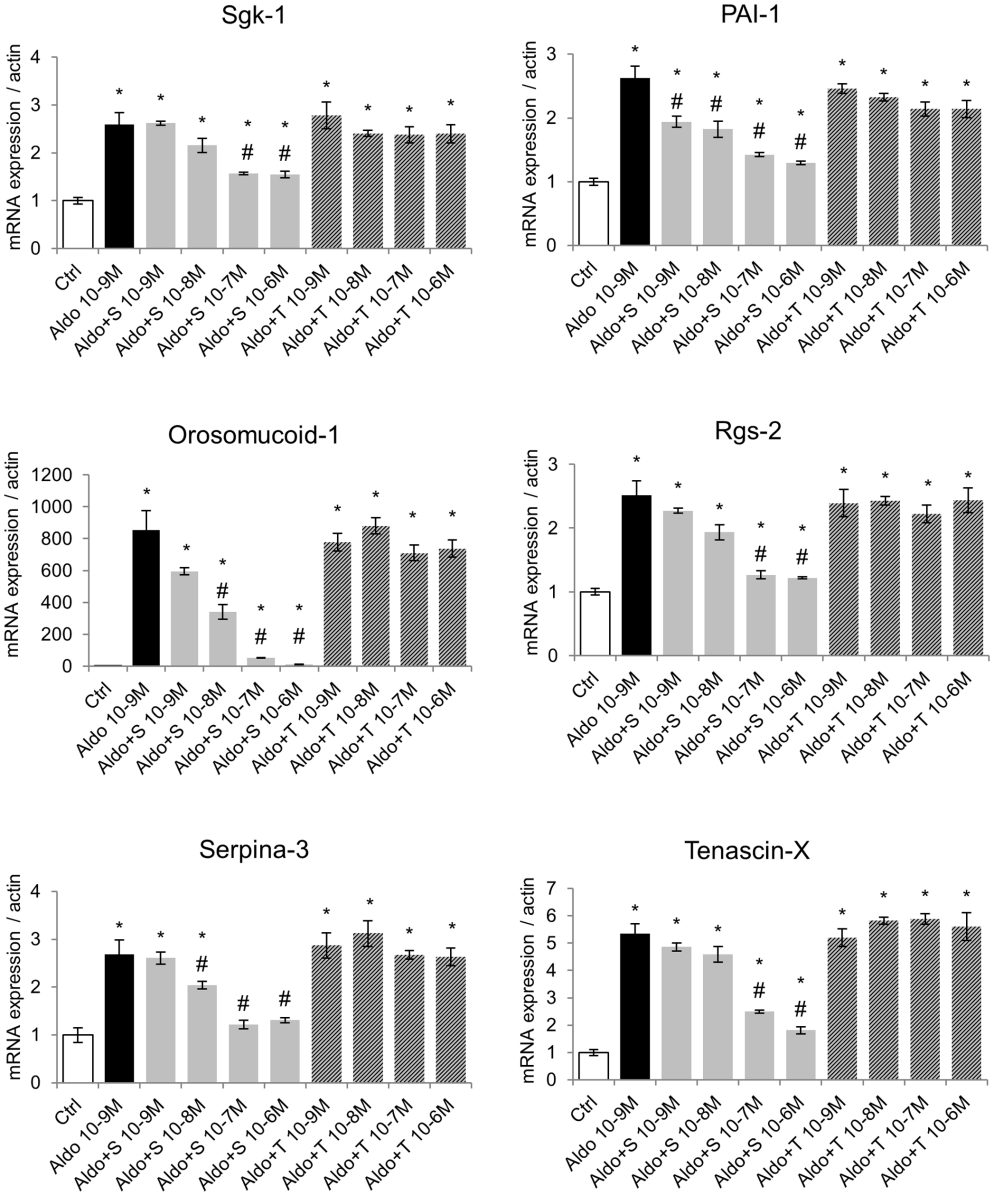
Torasemide does not act as a MR antagonist for the regulation of endogenous genes in H9C2-MR cells in the presence of 10^−9^ M aldosterone. 10^−9^ M aldosterone (Aldo) increased expression of the aldosterone-targets genes Sgk-1, PAI-1, Orosomucoid-1, Rgs-2, Serpina-3 and Tenascin-X. Addition of increasing doses of spironolactone (A+S) inhibited aldosterone-induced gene expression. In contrast, increasing concentrations of torasemide (A+T) had no antagonistic effect. Mean ± SEM (n = 4). *p<0.05 *vs* control (Ctrl); # *p*<0.05 *vs* aldosterone.

The MMTV promoter, despite being classically used as a reporter of MR activation, is distinct from natural targets for the MR in cardiomyocytes. It could therefore be argued that activation/antagonism of MR may be different in a natural cell context. We took advantage of our previous work that identifies MR-modulated cardiac genes [21] to test the effect of torasemide, in comparison to spironolactone, in H9C2-MR cells. We analyzed the expression of the Serum- and glucocorticoid- inducible kinase-1, Sgk-1 (regulated by corticosteroid hormones in kidney cells), Orosomucoid-1 (an inflammatory protein), Serpina-3, PAI-1 and Tenascin-X (genes of the extra-cellular matrix remodeling) and Rgs-2 (Regulator of G protein signaling-2). Torasemide (10^−6^ M) alone significantly decreased the basal expression (in absence of serum and aldosterone) of Orosomucoid-1 and Tenascin-X, which is indeed very weakly expressed in cardiomyocytes in the absence of adosterone stimulation (Figure S2). We next tested the antagonist activity of torasemide. As depicted in Figure 3, in the presence of 10^−8^ M aldosterone, spironolactone partly or fully inhibited aldosterone-induced expression of aldosterone/MR target genes while torasemide had no effect, even at a high concentration (10^−6^ M). Similar effects were observed when a lower concentration of aldosterone was used (10^−9^ M) (Figure 4) indicating that the absence of antagonist activity of torasemide was not due to unfavorable competition with a high aldosterone concentration. Interestingly, the potency of spironolactone varies among the analyzed target genes: PAI-1, Orosomucoid-1 and Serpina-3 were inhibited with 10 fold lower spironolactone concentration (10^−8^ M) than Sgk-1, Rgs-2 and Tenascin-X (Figure 4). We next evaluated whether a torasemide (10^−6^ M) could enhance the sensitivity of MR toward spironolactone in aldosterone-treated H9C2-MR cells. To this purpose, we treated cells with a low dose of spironolactone (10^−8^ M) that was not sufficient to reduce -the increase of gene expression with 10^−8^ M aldosterone. Addition of torasemide did not sensitize the MR to spironolactone as shown by the analysis of aldo-induced gene expression of Orosomucoid-1, Sgk-1 and Tenascin-X (Figure S2).

## Discussion

Spectacular progress has been made in the treatment of heart failure during the last decades. Combining drugs of different therapeutic classes such as diuretics, beta-blockers, Angiotensin Converting Enzyme inhibitors and Angiotensin receptor blockers led to major clinical benefits on morbidity-mortality. Despite these progresses, heart failure remains a life-threatening disease, requiring further improvement of the therapeutic strategies. Blocking the mineralocorticoid receptor has been proposed and validated as an additional tool: clinical studies in patients with severe or mild heart failure demonstrated an additional benefit of MRAs administered on the top of standard therapy [8]–[10]. The results of these trials led to changes in the therapeutic guidelines [22], [23]. One of the proposed underlying mechanisms explaining this clinical benefit relies on the decrease of myocardial fibrosis. Indeed targeting cardiac fibrosis would impact the severity of heart disease by reducing stiffness, improving cardiac hemodynamics and reducing pro-arrhythmic triggers. Cardiac anti-fibrotic therapy includes MRAs (such as spironolactone or eplerenone) and, more recently, the diuretic torasemide. Whether MRAs and torasemide act on the same pathways or not needs to be carefully addressed. Indeed if these therapeutic classes act on different pathways, it may be useful to use both of them which may lead to additive beneficial effects.

It has been proposed that torasemide has some mineralocorticoid receptor antagonist properties and torasemide is considered as an “anti-aldosterone” compound [5]. In order to test the potential MR antagonist property of torasemide, we selected several genes of different functional classes (inflammation, extracellular matrix remodelling, signalling) that were previously shown, by ourselves and by others, to be regulated by aldosterone in cardiomyocytes [16], [21]. Rgs-2 (Regulator of G protein signaling-2) is a multifunctional signaling regulator and exerts GTPase-activating protein activities. Many signals that regulate cardiomyocyte growth, differentiation, and function are mediated via heterotrimeric G proteins, which are controlled by Rgs proteins [24]. Three aldosterone-regulated genes have close connections with the regulation of the extra-cellular matrix: Plasminogen Activator Inhibitor-1 (PAI-1), a member of the SERPIN family, is the major physiological inhibitor of tissue-type Plasminogen Activator and Urokinase Plasminogen Activator, that activate plasminogen to its active form plasmin and lead to fibrinolysis. In addition to its serine protease inhibitor function, PAI-1 also alters cell/matrix interactions by binding to vitronectin [25]; Serpina-3 (also named α1-antichymotrypsine) is a serine protease inhibitor with multiple functions including maturation of pro-MMP9 and wound healing, regulation of apoptosis and inflammation [26]; Tenascin-X belongs to the family of matricellular proteins. These proteins are extracellular matrix proteins that modulate cell-matrix interactions and collagen accumulation and organization, but do not have a direct structural role [27]. Tenascin-X is an essential regulator of collagen deposition by fibroblasts [28] and is up-regulated during fibrosis after tissue injury [27]. Orosomucoid-1 is an acute phase protein that is induced by pro-inflammatory cytokines and glucocorticoids [29]. Interestingly, elevated plasma level of Orosomucoid-1 is considered as a cardiovascular risk factor [30].

The data reported in the present study clearly do not support the concept that torasemide acts as an antagonist of the MR. Indeed using different approaches, we showed that torasemide does not behave as spironolactone, a prototypic MRA: it did not affect nuclear translocation of MR nor inhibited aldosterone-induced up-regulation of various mineralocorticoid receptor target genes in cardiomyocytes. These results are not compatible with the study of Uchida et al. [5] proposing that torasemide acts as a competitive antagonist of aldosterone binding in kidney homogenates. A tissue-specific effect of torasemide on MR binding in kidney but not cardiomyocyte is a possibility but, to our knowledge, such tissue-specific actions of MR antagonists have not been reported.

From our results and previously published data, spironolactone not only competes with aldosterone for binding to MR, but it also induces ligand-dependent conformational changes that uncover nuclear localization signals, eliciting MR trafficking to the nucleus. The slower kinetics of this process when compared to aldosterone-induced nuclear translocation may reflect differences in ligand binding kinetics and ligand-induced co-chaperone release from the aporeceptor complex [31]. Therefore, spironolactone is not a classic competitive antagonist of MR. It has been proposed that the inhibitory effect of spironolactone on MR is based on the induction of an altered receptor conformation, which in turn disrupts co-activator recruitment [32]. This could be consistent with recent data demonstrating that spironolactone behaves as an inverse agonist of MR [33], [34] suggesting that spironolactone beneficial therapeutic actions involve the induction of MR-dependent effects that are opposite to those induced by natural agonists such as aldosterone. The molecular basis for the inverse agonist activity is unknown. It has been proposed that spironolactone can act as an MR agonist in a cell- and promoter-specific fashion [35], but the identities of specific target genes that may be regulated by spironolactone-MR complexes remain to be elucidated.

Torasemide, at micromolar concentrations, has also been reported to inhibit aldosterone secretion in vitro by adrenal cells from rats, cows and guinea pig upon stimulation by potassium and AngII (among other classical aldosterone secretagogues), while furosemide has no effect on adrenal aldosterone secretion [36]. In human, torasemide has been reported to inhibit transcardiac extraction of aldosterone in patients with congestive heart failure [37], an effect which might be due to inhibition of local aldosterone synthesis, that has been reported to be increased in human heart failure [38]. *In vivo* however, in both human and dogs, torasemide, as other diuretics, increases plasma aldosterone levels to compensate sodium loss [39], [40], [41]. Therefore, if MR is not properly antagonized, torasemide administration would likely increase MR activation through the rise in plasma aldosterone levels rather than decrease MR-mediated pathways.

Both torasemide and spironolactone have been shown to be efficient in patients and in dogs with HF [1], [42]. One can speculate that adding torasemide to spironolactone in HF would be beneficial. Torasemide has a strong diuretic effect and an anti-fibrotic effect for which a mechanism has been recently proposed: torasemide inhibits procollagen type I carboxy-terminal proteinase activation as well as lysyl oxidase expression and collagen cross-linking in patients with heart failure [11], [12], resulting in normalization of left ventricular stiffness. MRAs act at different levels: MRAs prevent electrophysiological abnormalities, oxidative stress and extracellular matrix remodeling as well as inflammation [7]. Beneficial additive effects may therefore occur in patients. Such therapeutical benefit in HF remains to be addressed.

## Perspectives

Taken together, these results demonstrate that the diuretic torasemide does not act as a mineralocorticoid receptor antagonist as previously proposed. This suggests that the use of torasemide together with an MR blocker could further enhance the anti-fibrotic effects of these therapeutics, especially in heart failure. This should be particularly considered since torasemide increased aldosterone, the ligand of the MR, due to its effects of the renal Na balance. This should be analyzed in dedicated experimental and clinical studies.

## Supporting Information

Figure S1 **Effect of Torasemide on Ligand-dependent transactivation activity of MR in H9C2-MR cells.** 10^−9^ M aldosterone (Aldo) increased MR transactivation activity, which was fully inhibited by the MR antagonist spironolactone (S) at 10^−7^ M while torasemide (T) has no effect. Mean ± SEM (n = 4). *p<0.05 *vs* control (Ctrl); # *p*<0.05 *vs* aldosterone.
(TIF)

Figure S2 **Torasemide did not enhance MR sensitivity to a low dose of spironolactone for the regulation of endogenous genes in H9C2-MR cells.** A low dose of spironolactone (10^−8^ M) did not block aldosterone-induced response of Orosomucoid-1, Sgk-1 and Tenascin-X. Torasemide (10^−6^ M) did not confer higher sensitivity to the spironolactone antagonist when spironolactone and torasemide were combined. Mean ± SEM (n = 4). *p<0.05 *vs* control (Ctrl).
(TIF)

Table S1 **Sequences of the specific primers.** Actin: β-actin; PAI-1: Plasminogen Activator Inhibitor-1; Rgs-2: Regulator of G protein signaling-2; Sgk-1: Serum- and glucocorticoid-inducible kinase-1.
(TIF)
