# Supplementary figures and images for: The Diuretic Torasemide Does Not Prevent Aldosterone-Mediated Mineralocorticoid Receptor Activation in Cardiomyocytes
Source: PLoS One. 2013 Sep 9;8(9):e73737. doi: 10.1371/journal.pone.0073737 (PMC3767808; doi:10.1371/journal.pone.0073737)

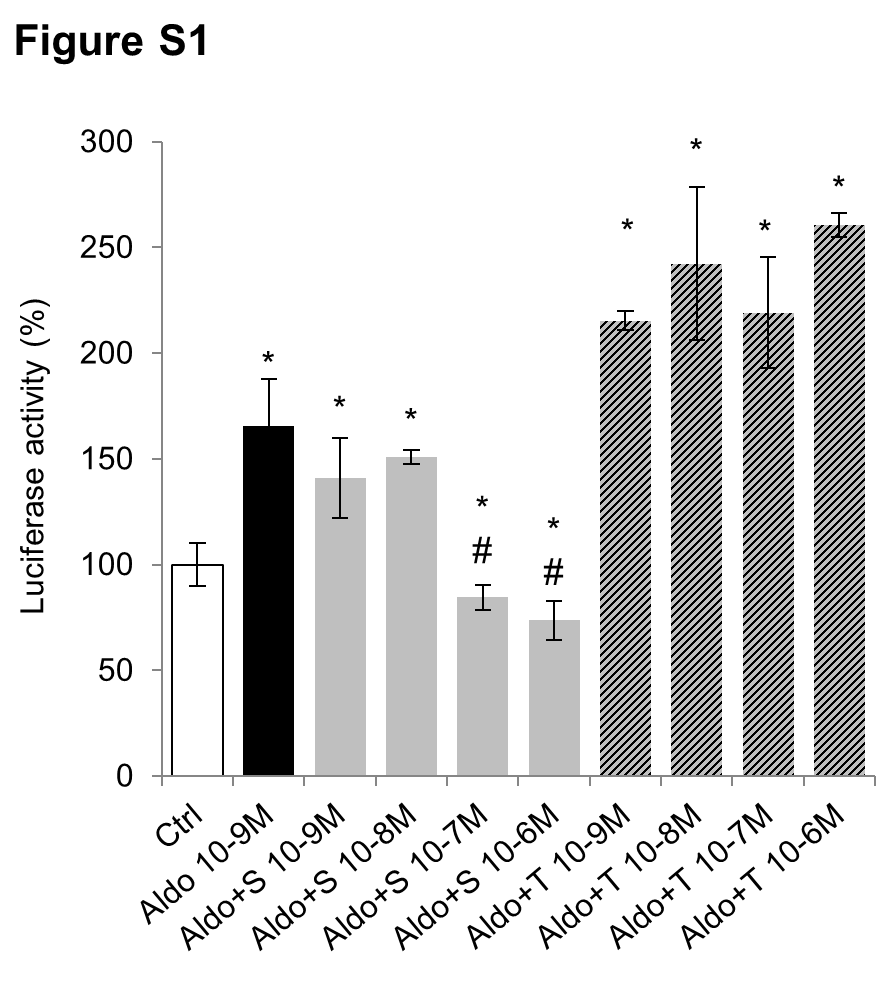

Supplement: Figure S1 — Effect of Torasemide on Ligand-dependent transactivation activity of MR in H9C2-MR cells. 10−9 M aldosterone (Aldo) increased MR transactivation activity, which was fully inhibited by the MR antagonist spironolactone (S) at 10−7 M while torasemide (T) has no effect. Mean ± SEM (n = 4). *p<0.05 vs control (Ctrl); # p<0.05 vs aldosterone. (TIF) [file pone.0073737.s001.tif]

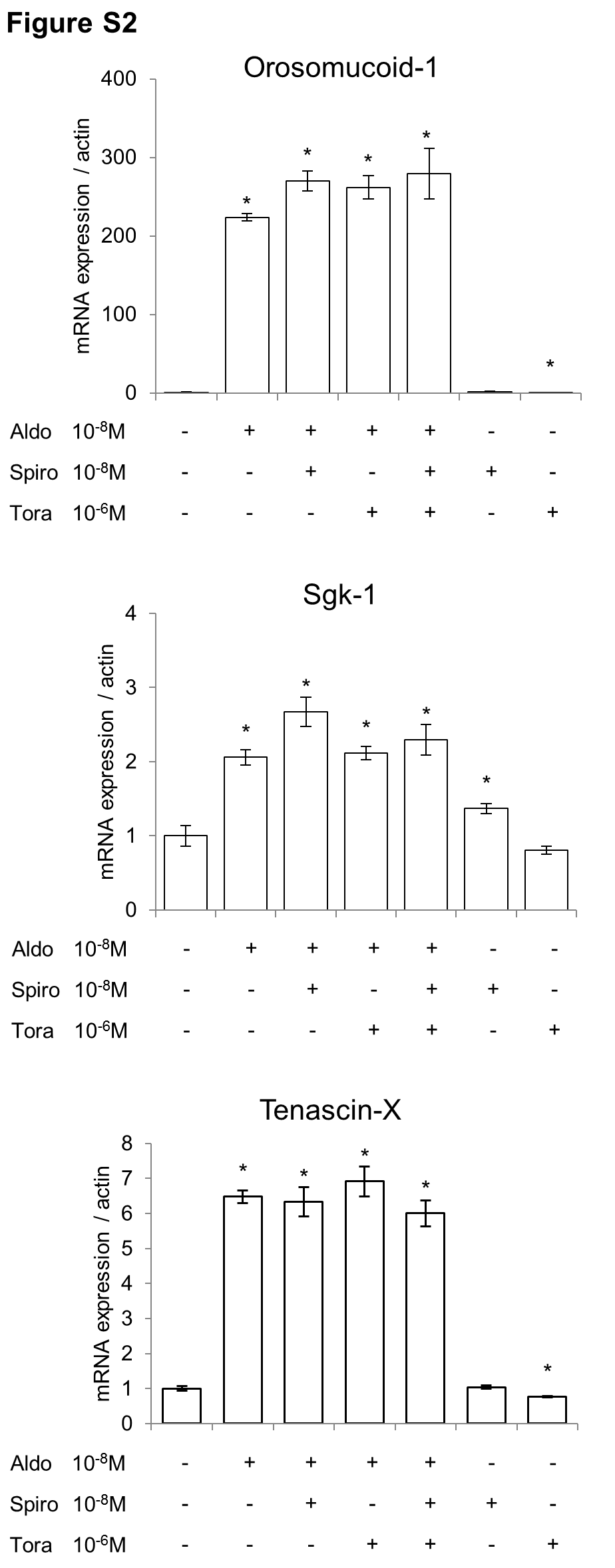

Supplement: Figure S2 — Torasemide did not enhance MR sensitivity to a low dose of spironolactone for the regulation of endogenous genes in H9C2-MR cells. A low dose of spironolactone (10−8 M) did not block aldosterone-induced response of Orosomucoid-1, Sgk-1 and Tenascin-X. Torasemide (10−6 M) did not confer higher sensitivity to the spironolactone antagonist when spironolactone and torasemide were combined. Mean ± SEM (n = 4). *p<0.05 vs control (Ctrl). (TIF) [file pone.0073737.s002.tif]

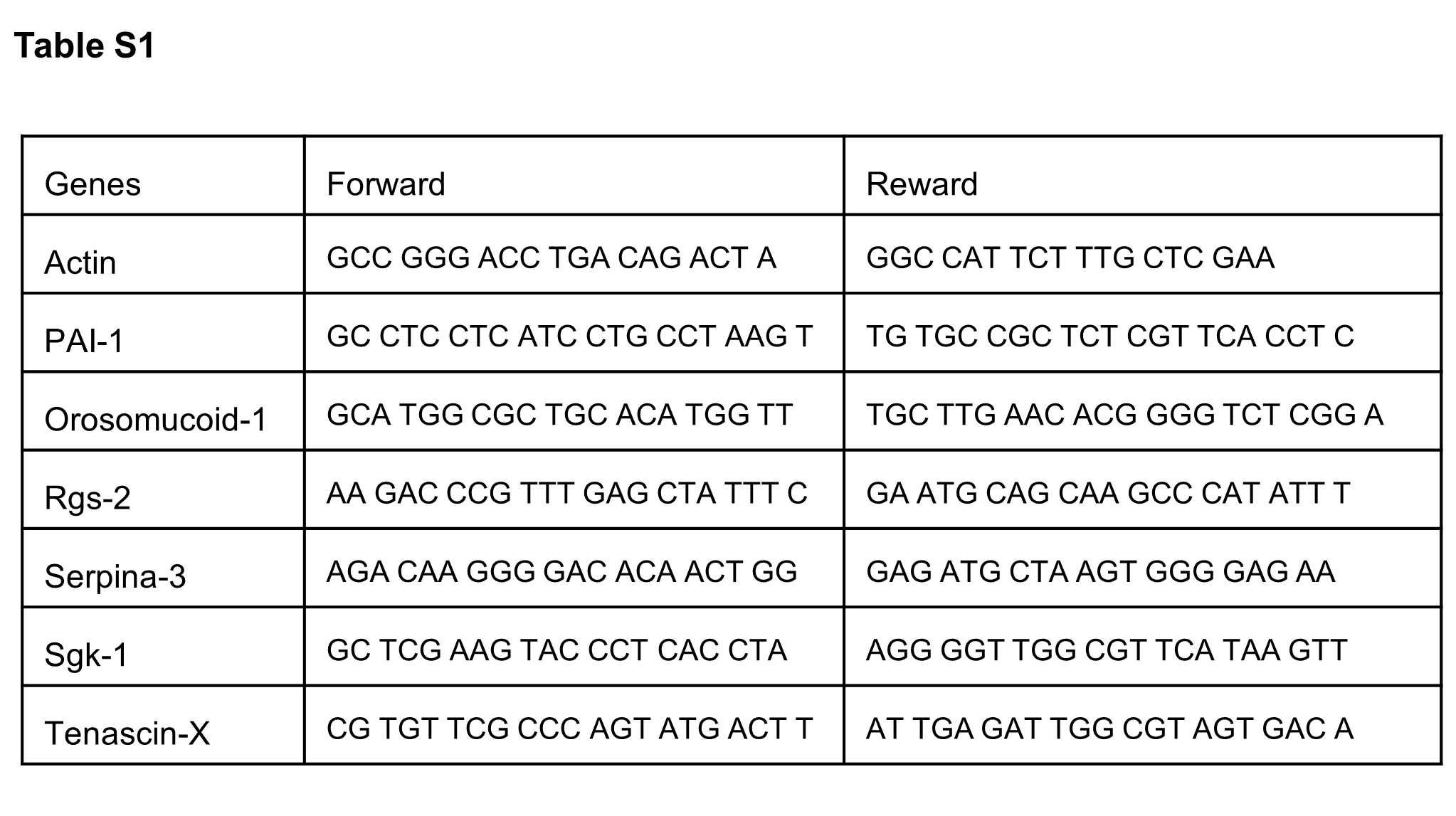

Supplement: Table S1 — Sequences of the specific primers. Actin: β-actin; PAI-1: Plasminogen Activator Inhibitor-1; Rgs-2: Regulator of G protein signaling-2; Sgk-1: Serum- and glucocorticoid-inducible kinase-1. (TIF) [file pone.0073737.s003.tif]
